# Sensitive, Stable, and Recyclable ZnO/Ag Nanohybrid Substrates for Surface-Enhanced Raman Scattering Metrology

**DOI:** 10.1021/acsmaterialsau.4c00002

**Published:** 2024-04-10

**Authors:** Samriti, Promod Kumar, A. Yu. Kuznetsov, H. C. Swart, Jai Prakash

**Affiliations:** † Department of Chemistry, National Institute of Technology Hamirpur, Hamirpur, Himachal Pradesh 177005, India; ‡ Department of Physics, University of the Free State, Bloemfontein 9301, Republic of South Africa; § Department of Physics, Centre for Materials Science and Nanotechnology, University of Oslo, Oslo N-0316, Norway

**Keywords:** nanoparticles, embedded nanocrystals, interfaces, ZnO, surface-enhanced Raman scattering (SERS) metrology, multifunctional application

## Abstract

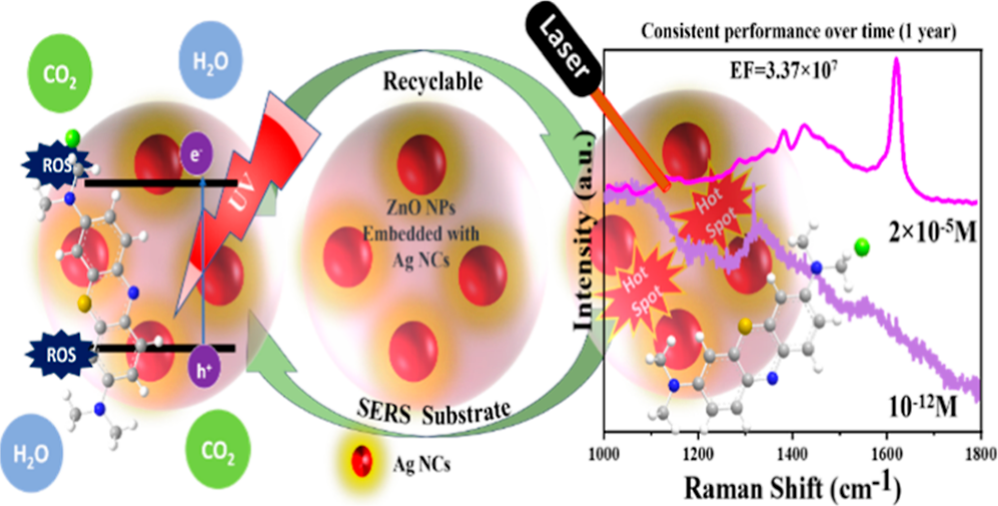

Surface-enhanced Raman scattering is a practical, noninvasive spectroscopic technique that measures chemical fingerprints for varieties of molecules in multiple applications. However, synthesizing appropriate substrates for practical, long-term applications of this method has always been a challenging task. In the present study, we show that ZnO/Ag nanohybrid substrates may act as highly stable, sensitive, and recyclable substrates for surface-enhanced Raman scattering, as illustrated by the detection of methylene blue, selected as a test dye molecule with self-cleaning functionalities. Specifically, we demonstrate the detection enhancement factor of 3.7 × 10^7^ along with exceptional long-term stability explained in terms of the localized surface plasmon resonance from the Ag nanocrystals embedded into the chemically inert ZnO nanoparticles, constituting the nanohybrid. Significantly, these substrates can be efficiently cleaned and regenerated while maintaining their high performance upon recycling. As a result, using these substrates, up to 10^–12^ M detection sensitivity has been demonstrated, enabling the accuracy required in modern environmental monitoring, bioassays, and analytical chemistry. Thus, ZnO nanoparticles with embedded Ag nanocrystals constitute a novel class of advanced nanohybrid substrates for use in multiple applications of surface-enhanced Raman scattering metrology.

## 1 Introduction

Raman spectroscopy is well-known for its capability to identify materials using vibrational fingerprints. However, classical Raman spectroscopy operates with relatively low sensitivity and, as such, has limited applicability for the identification of analyte molecules exhibiting low concentrations.^1^ In its turn, surface-enhanced Raman scattering (SERS) has become an effective spectroscopic method for ultrasensitive detection and examination of analyte molecules. SERS is able to increase Raman spectroscopic signals by several orders of magnitude because of the localized surface plasmon resonance (LSPR), pushing the detection limit and allowing the identification of even very low traces of analytes.^1,2^ As such, SERS has been used in various fields on the account of its quick response, nondestructive nature, real-time detection, and ultrasensitive characteristics.^1−3^ In accordance with the literature, the SERS is a function of charge transfer and localized electromagnetic field enhancement processes acting in combination.^2^ In the charge transfer mechanism, the chemisorption of the analyte molecule leads to a change in the analyte molecule’s polarizability associated with modifications of the electronic structure. Thus, the charge transfer process that takes place between an analyte molecule and a substrate is particularly important for SERS enhancement on semiconductor substrates. The prime condition is that the energy levels/bands of the analyte/substrate must be properly aligned for the charge transfer to be carried out.^2^ On the other hand, the electromagnetic field mechanism is common when using noble metal substrates with LSPR characteristics. Nevertheless, the selection of highly sensitive SERS substrates also exhibiting high stability and recyclability is challenging.^2^ Concurrently recyclable SERS substrates emerged as important components for the environmental studies.^4−8^ Indeed, adsorbed molecules, ambient contaminants, or leftover analytes from earlier measurements affect SERS data due to interference, high background signals, etc.

By far, a range of wide bandgap semiconductors, specifically those complaining with cost-effectiveness and low toxicity criteria, have been tested for the SERS metrology.^6,7,9−11^ Particularly, metal-oxide semiconductors, e.g., ZnO and TiO_2_, were employed as efficient SERS substrates. These materials are also effective photocatalysts, and because of their self-cleaning properties, these oxides were suggested to be promising recyclable SERS substrates.^12−14^ Notably, ZnO exhibits a higher refractive index and stronger optical absorption, making it more efficient for SERS applications.^15−18^ Additionally, its surface has more electrochemically active sites than that of TiO_2_, which improves SERS signals and photocatalytic activity on its surface due to the charge transfer mechanism.^15,16,19^ In its turn, noble metal nanostructures, including Ag, Au, etc., have been used extensively as SERS substrates too, due to their excellent LSPR properties, featuring intense absorption and “hot spots”, resulting in an enhanced local electromagnetic field providing conditions for ultrasensitive SERS detection.^20,21^ Thus, the current state of the art is in designing heterogeneous composites combining the unique LSPR characteristics of the noble metals with the self-cleaning capabilities of the metal oxide nanostructures in order to produce highly sensitive and recyclable SERS substrates with tunable characteristics for long-term practical applications.^4,5,7,13,22,23^

For instance, Wang et al.^23^ recently demonstrated that ZnO nanorods decorated with Ag nanoflowers enabled ultrasensitive SERS detection and recyclability of dye molecules with a detection limit of 10^–13^ M. Similarly, Li et al.^24^ reported the self-cleaning SERS substrate consisting of Ag nanocrystals decorating ZnO nanorods, demonstrating an enhancement factor of 2.7 × 10^8^ and a detection limit of 10^–10^ M of rhodamine 6G dye. Chen et al.^25^ reported similar results for ZnO/Ag composite substrates, showing excellent sensitivity and the detection limit for another rhodamine dye too. However, in spite of these breakthrough high sensitivities reached with the use of the noble metal/semiconductor composites, the long-term stability and reproducibility are still of great concern in the SERS metrology,^13,26^ as such, motivating further efforts along this promising direction.

To enhance these parameters, instead of decorating ZnO nanostructures with Ag nanocrystals, we propose to investigate an alternative option of embedding Ag nanocrystals (NCs) into the nanosized ZnO nanoparticles (NPs), as such creating a novel type of the SERS substrates, denoted as ZnO/Ag nanohybrid in the rest of the paper, to distinguish it from the rest of previously studied composite SERS substrates. In this paper, we report a facile sol–gel fabrication method of such novel nanohybrid ZnO/Ag structures exhibiting excellent SERS sensitivity, recyclability, and long-term stability. This approach not only provides a cost-effective framework for the SERS metrology but also paves the way to increase the long-term stability with excellent SERS performance, utilizing the energy coupling at the interface between the embedded Ag NCs and ZnO NPs matrix.

## 2 Results and Discussion

The ZnO/Ag nanohybrids were synthesized using a simple sol–gel method by varying the concentration of Ag (0–20 mol %), followed by a calcination process at high temperature, i.e., 800 °C. The resulting ZnO/Ag nanohybrids are named 0, 1, 5, 10, 15, and 20 AZ as per increasing Ag concentrations of 0, 1, 5, 10, 15, and 20 mol %, respectively. Various characterizations were performed and are summarized in this section for a better understanding of their properties, such as structural and optical properties, as well as their chemical composition for further functional applications.

### 2.1 Characterization of ZnO/Ag Nanohybrids

**Figure 1:**
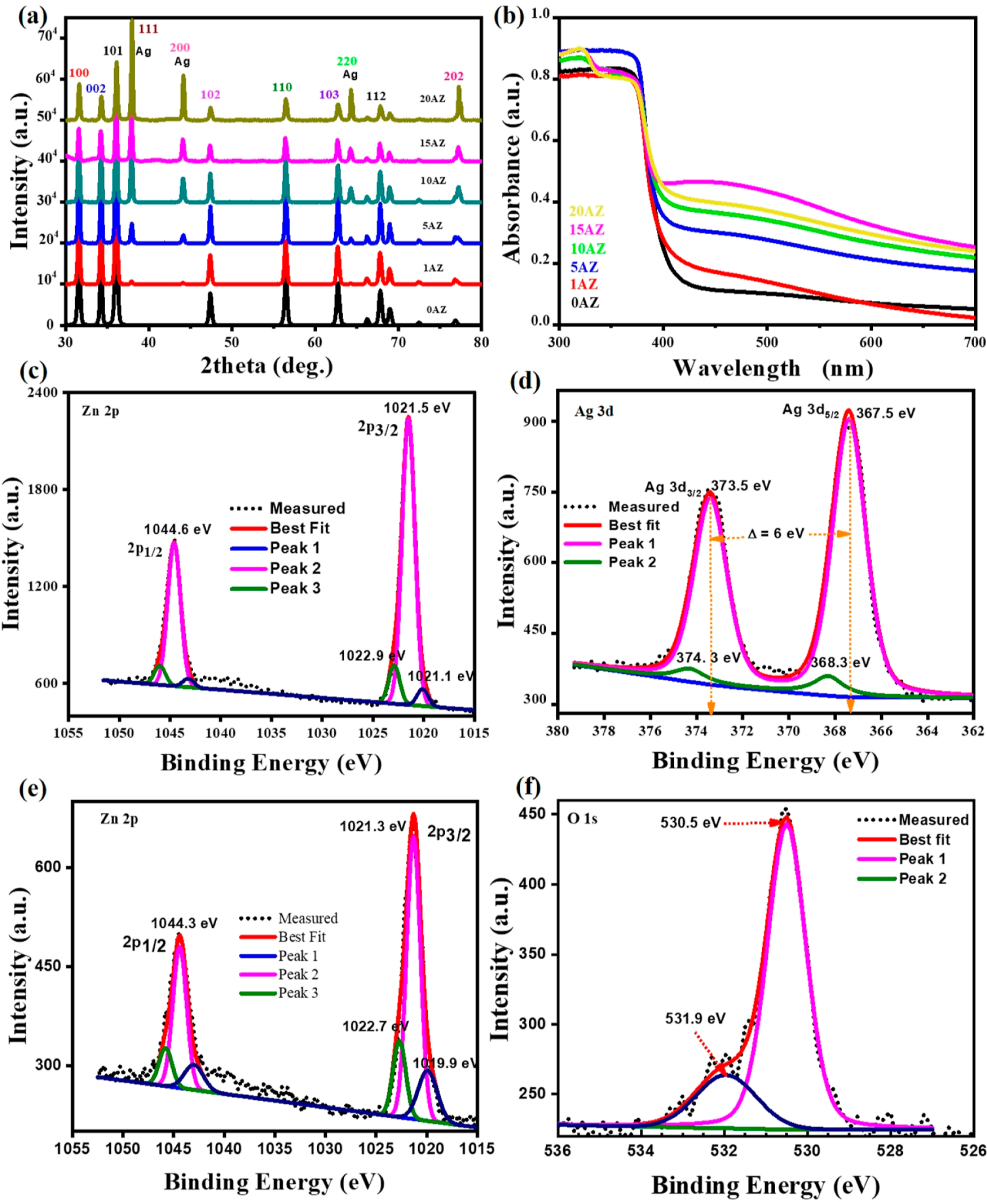
(a) XRD spectra of the ZnO/Ag nanohybrids by varying the Ag content (0–20 AZ). (b) Optical absorbance spectra of the host ZnO NPs and ZnO/Ag nanohybrid structures with different Ag contents. (c) Zn 2p XPS spectra of 0 AZ (d–f) XPS spectra of Ag 3d, Zn 2p, and O 1s of 15 AZ.

XRD patterns of the pure ZnO NPs (labeled as 0 AZ) and ZnO/Ag nanohybrids with different Ag contents in the range of 1–20 mol % (correspondently labeled as 1, 5, 10, 15, and 20 AZ) are shown in Figure 1a. The peak at 2θ = 36.07° corresponds to the (101) plane of ZnO, and 37.89° corresponds to the (111) plane of Ag. All these peaks are well indexed in the literature, representing the hexagonal wurtzite phase of ZnO (JCPDS card no. 36-1451) and the FCC phase of Ag (ICSD no. 52362).^9,27−30^ No additional peaks related to impurities were observed, indicating the high purity of the samples. It is seen that for ZnO/Ag nanohybrids, the (101) characteristic peak of the ZnO moves to lower angles (at lower Ag concentrations up to 15 AZ) when compared to pure ZnO (see in Figure S1a). It was attributed to the incorporation of Ag^+^ ions in the lattice of the host ZnO NPs, up to the solubility limit. Indeed, the host Zn^2+^ and Ag^+^ ions have ionic radii of 0.74 and 1.26 Å, respectively; therefore, there is a limit for Ag^+^ ions to replace Zn^2+^ in the ZnO lattice.^31,32^ According to Naskar et al.,^33^ Ag^+^ ions act as monovalent dopants, occurring in both substitutional and interstitial lattice sites of ZnO. Such dopant configurations are energetically favorable at lower Ag concentrations, while segregation of Ag into metallic form dominates at higher Ag concentrations.^31,34^ Further, the intensity of the peaks corresponding to metallic Ag also increases for the sample with increasing Ag concentration, resulting in the formation of Ag NCs.^9,28,31,35^ Such an intensity increase of the Ag(111) peak suggests that the Ag NCs develop a more ordered and well-defined crystalline structure, whereas the ZnO host NPs may exhibit a lower degree of crystallinity. Similar results were observed by Gupta et al.,^31^ in the case of Ag-embedded ZnO nanocomposites. Indeed, similar to our work, Gupta et al.,^31^ confirmed the formation of Ag NCs by observations of the comparable shifts in the XRD peak with Ag doping and an increase in the intensity of the Ag(111).Similarly, Ha Pham et al.^36^ and Alharthi et al.^37^ corelated the formation of Ag NCs with increasing Ag content; Ag(111) diffraction peak intensity increases as a function of Ag introduction into ZnO NPs, interpreted as the formation of the Ag–ZnO nanocomposites.

The optical absorption of ZnO/Ag nanohybrids is shown in Figure 1b as a function of Ag content. At 370 nm, a sharp band edge is consistent with the ZnO host NPs bandgap absorption. Notably, a minor shift in the position of the ZnO absorption edge is observed as a function of the Ag content and attributed to the changes in morphology, particle size, and surface microstructure.^38^ Importantly, a distinct peak emerges at 471 nm for Ag (1 AZ sample), corresponding to the LSPR absorption by Ag NCs. The intensity of this peak gradually increases with increasing Ag concentration up to 15 AZ and then decreases.^16,39^ Thus, for 20 AZ, aggregation or clustering of Ag NCs is more likely to occur. Furthermore, for the 20 AZ sample, a red shift is also observed, potentially because the interparticle distance decreases as the Ag concentration increases, resulting in a greater electromagnetic interaction between the Ag NCs. The absorption edge may shift toward longer wavelengths as a result of this coupling, which may cause a red shift in the plasmon resonance frequency.^40^

The Mie theory formula *d* = 2*R* = 2 was used to calculate the size of Ag NCs.^41,42^

Where ℏ is Planck’s constant, υ_F_ is the Fermi velocity of Ag NPs (1.39 × 10^6^ m/s), and *d* is the average diameter of the Ag NCs. The energy distribution Δ*Ε* was estimated from the full width at half-maximum of the plasmonic band of the Ag NCs. With increasing Ag concentration up to 20 AZ, the average size of the Ag NCs was determined to be 2.3, 3, 3.5, 1.4, and 1.5 nm using the Gaussian profile from the UV–vis spectra of the Ag NCs (Table S1, Supporting Information). According to this analysis, the size of Ag NCs increases from 1 to 10 AZ samples. The Ag NCs are far apart and grow with an increasing Ag concentration. For 15 AZ, the size of Ag NCs decreases in spite of the increased density of the NCs (consistent with HRTEM data shown later) corelated with the LSPR enhancement in the absorption spectra (Figure 1b).^43,44^ The enhanced intensity of LSPR could potentially be attributed to the optimized interparticle distance and effective coupling of LSPR in closely positioned Ag NCs embedded within the host ZnO NPs.^20^ This optimized interparticle distance allows for excellent LSPR performance in our structure, which we call “nanohybrid” to distinguish it from previously used nanocomposites. For 20 AZ, even though the size of Ag NCs is comparable to that of 15 AZ (both by Mie calculation and HRTEM), the density of Ag NCs increases too, causing a decrease in the LSPR.

XPS was performed to evaluate the chemical states of the different elements in the 0 and 15 AZ samples, as shown in Figure 1c–f respectively. For the host ZnO NPs, the binding energy envelope of Zn 2p is divided into two major peaks at 1021.5 and 1044.6 eV. The former one is further divided into three peaks at 1020.1, 1021.5, and 1022.9 eV; see Figure 1c. These peaks are related to different chemical types of Zn chemical states. The peak recorded at 1021.5 eV is the stoichiometric ZnO; the peak at 1022.9 eV is related to Zn(OH)_2_, and the peak at 1020.1 eV may be correlated with broken ZnO bonds or impurities.^45^ Similarly, Zn 2p fits were performed for the Zn part of the spectra in other samples containing Ag NCs (not shown here). Ag is characterized by its Ag 3d spectrum resolving two peaks at 367.5 and 373.5 eV, corresponding to Ag 3d_5/2_ and Ag 3d_3/2_, respectively, confirming the Ag metallic state (Figure 1d). The peak at 367.5 eV is fitted with two components: a major peak at 367.5 eV and a minor peak at 368.3 eV, which shows that some fraction occurred in a different configuration presumably in the form of Ag_Zn_.^31^ Importantly, no peaks corresponding to AgO and Ag_2_O were observed, confirming that the majority of the Ag NCs are embedded into host ZnO NPs in metallic form. Similarly, Gupta et al.^31^ prepared Ag-embedded ZnO nanocomposite and observed a similar peak at 367.5 eV and a very minor one at 368.2 eV. This result suggested that a small number of Ag NPs were formed and entered interstitials, while the majority of Ag travels as Ag^+^ ions and occupies Zn sites (Ag_Zn_).

Furthermore, the Zn 2p spectrum of Ag is shown in Figure 1e, with two main peaks at 1021.3 and 1044.3 eV. Figure 1f displays the XPS spectra of O 1s of 15 AZ with two peaks at 530.5 and 531.9 eV. The peak at 530.5 eV corresponds to the crystal lattice oxygen in ZnO.^46^ A small peak at 531.9 eV is attributed to surface contaminants such as AgO, hydroxides, and other surface contaminants on Ag/ZnO, as reported in the literature.^47,48^

**Figure 2:**
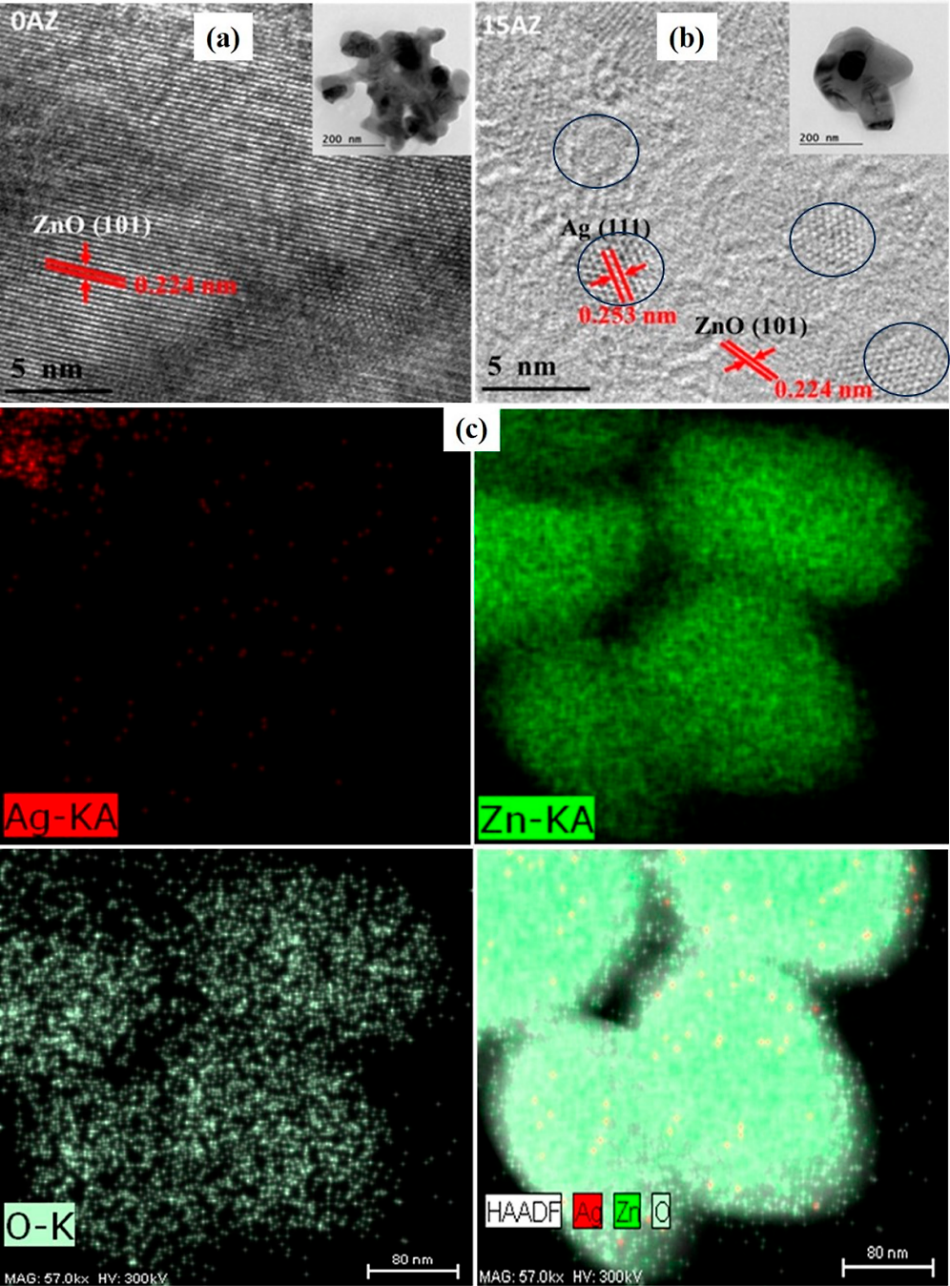
(a,b) TEM (inset) and HRTEM images of 0 and 15 AZ. (c) Elemental mapping of Ag (red), Zn (green), and O (light green) and ZnO/Ag nanohybrid.

The ZnO/Ag nanohybrids were further examined by TEM and HRTEM to confirm that the Ag NCs were indeed embedded in the host ZnO NPs. The TEM images of the 0 and 15 AZ samples, as shown in Figure S2a,b, demonstrate their porous nanostructure, which allows for better adsorption of analyte molecules.^49−51^ Furthermore, the BET studies also reveal that 0 and 15 AZ had a high surface area (49.5 and 24.4 m^2^/g respectively) and pore sizes of 21.7 and 16.6, respectively, which confirm their porous nature. Figure 2a,b show HRTEM images of the 0 and 15 AZ samples. The (101) and (111) planes of ZnO and Ag, represent lattice fringe spacings of 0.224 and 0.253 nm, respectively,^52,53^ which are consistent with the XRD pattern in Figure 1a. Thus, Ag NCs are embedded into the host ZnO NPs, as visualized in the HRTEM image of Figure 2b, which is in consistent literature, as discussed above in XPS details. Similarly, Figures S3 and S4 show the HRTEM images and size distribution of the 10 and 20 AZ samples with embedded Ag NCs, confirming the size and density trends for Ag NCs in accordance with the Mie calculations. Furthermore, TEM images of the 0 and 15 AZ samples are also shown in the insets of Figure 2a,b, respectively, which demonstrate the absence of any Ag NCs on the surface of host ZnO NPs in the 15 AZ samples, realizing the fact that Ag NCs may be embedded in ZnO NPs. These samples were further analyzed with FESEM and EDS mapping (large area from FESEM) to confirm the absence of Ag in the 0 AZ sample and the presence of Ag in the 15 AZ sample, as shown in Figure S5. Furthermore, for more clarity and better understanding of Ag embedded ZnO (15 AZ) NPs, the distribution of Ag, Zn, and O in the 15 AZ sample (small area from TEM image) is measured by elemental mapping, as shown in Figure 2c, i.e., elemental mapping for Ag (red), Zn (green), and O (light green). It clearly shows that Zn and O have larger densities than Ag. As a result, elemental mapping reveals high purity and uniformity, confirming the nanohybrid structure of Ag NCs embedded into the host ZnO NPs. Moreover, the FTIR, TGA, and Raman spectra of ZnO/Ag nanohybrid (0, 15 AZ) are consistent with the above-discussed results (briefly explained in Supporting Information Figure S6).

### 2.2 SERS Detection of Methylene Blue Molecules

Methylene blue is a basic (cationic) dye with numerous uses in the leather, cosmetic, pharmaceutical, and textile industries. There is a great demand for ultrasensitive detection and degradation of methylene blue and similar dyes.^54−56^ Thus, we tested the ZnO/Ag nanohybrid as SERS substrates with a self-cleaning functionality to satisfy the methylene blue detection/degradation criteria and discovered its high stability, recyclability, and high sensitivity.

**Figure 3:**
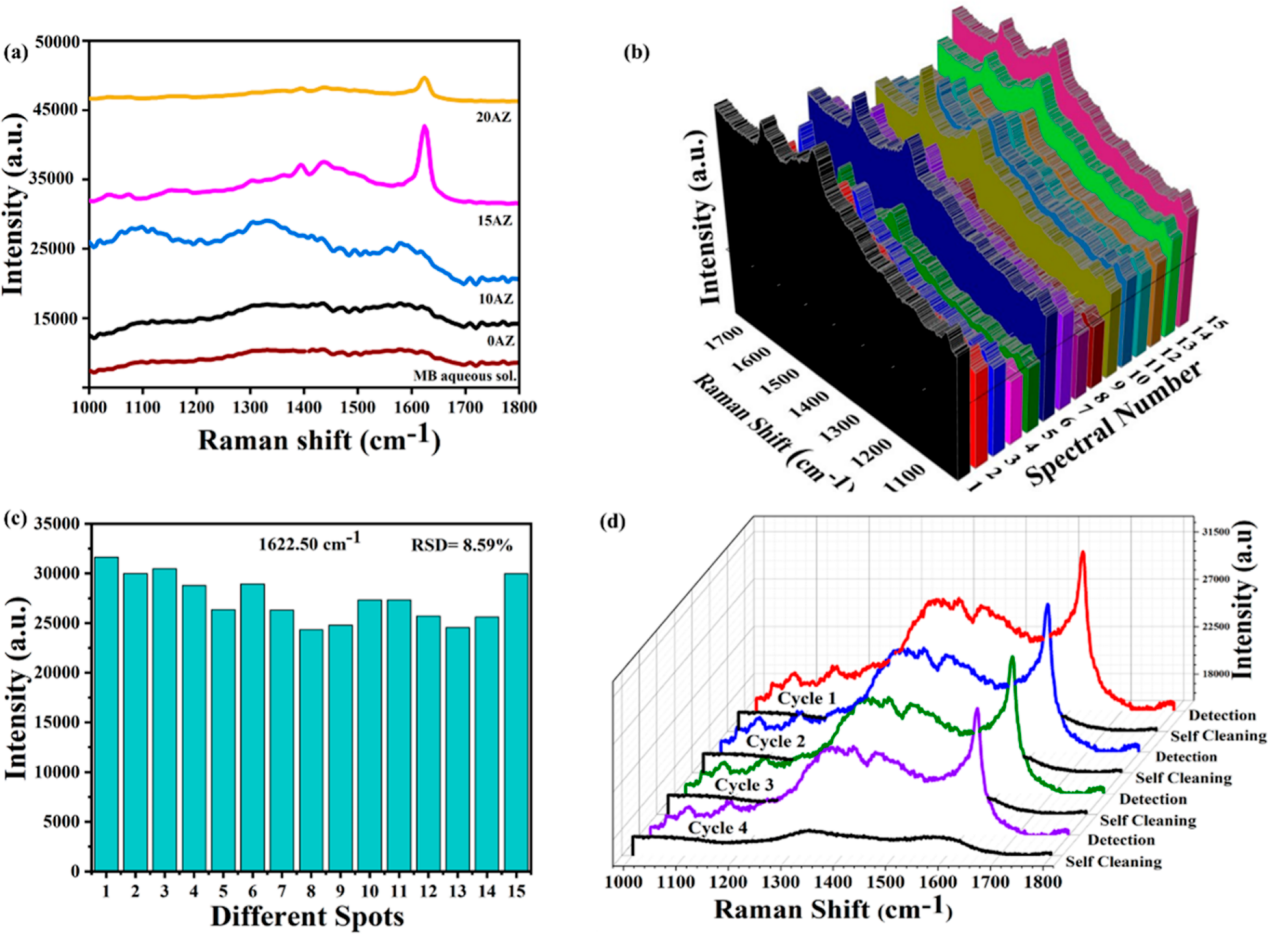
(a) SERS spectra of an aqueous solution of methylene blue dye adsorbed on ZnO/Ag nanohybrids (0–20 AZ). (b) SERS spectra of methylene blue dye collected from 15 different spots on the 15 AZ sample. (c) Distribution of the relative signal intensities for the 1622.50 cm^–1^ peak in the 15 AZ sample. (d) SERS spectra for recyclability tests in a 15 AZ sample under four degradation cycles (adsorption of methylene blue, followed by UV irradiation for 120 min).

The normal Raman spectra of MB in powder form and MB in aqueous solution are displayed in Figure S7. It is evident that the low concentration of MB molecules is the reason for the less pronounced Raman signals of MB in aqueous solution. Figure 3a shows the SERS spectra of methylene blue molecules adsorbed on ZnO/Ag nanohybrid substrates in a 2 × 10^–5^ M aqueous solution. The weak peaks at 1622.50 and 1328 cm^–1^ originate from the C–C ring stretching and C–N stretching modes in the methylene blue molecule, respectively. Apparently, without substrate, the Raman signals are weak. However, the ZnO/Ag nanohybrid enhances SERS signals for the methylene blue molecules adsorbed on the ZnO/Ag nanohybrid substrate surface. As illustrated in Figure 3a, the 0 AZ sample has little effect on the Raman signal enhancement of the methylene blue. However, SERS of methylene blue adsorbed onto ZnO/Ag nanohybrid substrates showed a considerable increase in SERS signal strength with increasing Ag content. This is anticipated because decreases in particle size, an increase in Ag NC density in ZnO NPs, and optimized internuclear distance between Ag NPs (discussed in UV spectra) are likely to result in more “hot spots”, which will boost SERS activity.^20^ By using the 1622.50 cm^–1^ peak as the index, it is evident that ZnO/Ag nanohybrid greatly boosted Raman peak intensity, showing a maximum for the 15 AZ sample, which is attributed to the strong LSPR results from the increase in the number of hot spots, as discussed above.^20^ It can be explained based on the electromagnetic and charge transfer mechanism enhancements of the SERS signals.^57^ Moreover, when two Ag NCs approach each other, the electromagnetic field is magnified dramatically due to numerous SERS hot spots.^57^ Apart from that, the 0 AZ sample shows a minor enhancement in the SERS signal, which may be attributed to the charge transfer of electrons from the ZnO to the LUMO of the methylene blue molecule. This charge transfer process can lead to changes in the vibrational properties of the molecule, resulting in an enhanced Raman scattering signal.^2^ Samriti et al.,^10^ observed similar results in the case of pure TiO_2_ nanorods for the detection of methylene blue molecules because of better charge transfer, leading to enhanced SERS signals, consistent with the review by Samriti et al.^2^

Validating the reproducibility of SERS signals and substrate stability is necessary to obtain a good and multifunctional SERS platform. Figure 3b shows the SERS mapping of the adsorbed methylene blue molecules on the 15 AZ substrates, demonstrating the good reproducibility of the substrate over the entire area (data were taken at several locations). The relative standard deviation (RSD) of the characteristic peak at 1622.50 cm^–1^ is found to be 8.59% (Figure 3c), confirming the high reproducibility of the SERS substrate (measurements using 15 AZ samples). Furthermore, methylene blue molecules were entirely adsorbed prior to testing, maintaining a constant methylene blue concentration on the surface of the samples. These ZnO/Ag nanohybrids possess a great potential to become highly useful substrates for real-world applications, e.g., competing with recyclable Ag-deposited TiO_2_ flower-like composite, as recently demonstrated by Yang et al.^22^ malachite green dye.

To study the recyclability and self-cleaning ability of the ZnO/Ag nanohybrids (15 AZ) sample, the characteristic Raman peak (1622.50 cm^–1^) was defined as the marker peak for tracking the photocatalytic degradation reaction. Following SERS detection, the SERS substrates were kept for 2 h under a UV lamp (385 nm) for methylene blue photodegradation. Before determining whether photocatalytic degradation was complete, the cleanliness of the substrates was checked using SERS detection. The same procedure was repeated for four consecutive cycles to evaluate the stability and recyclability of the material. Figure 3d indicates exceptional stability, as there is no discernible shift in the marker peaks before and after the recyclability test. The total decrease in SERS activity after 4 cycles was limited to 10.19% only, which indicates good stability. The benefit of photocatalytic degradation is that it breaks down methylene blue molecules into CO_2_ and H_2_O, giving the SERS substrates the ability to serve as self-cleaning substrates.

**Figure 4:**
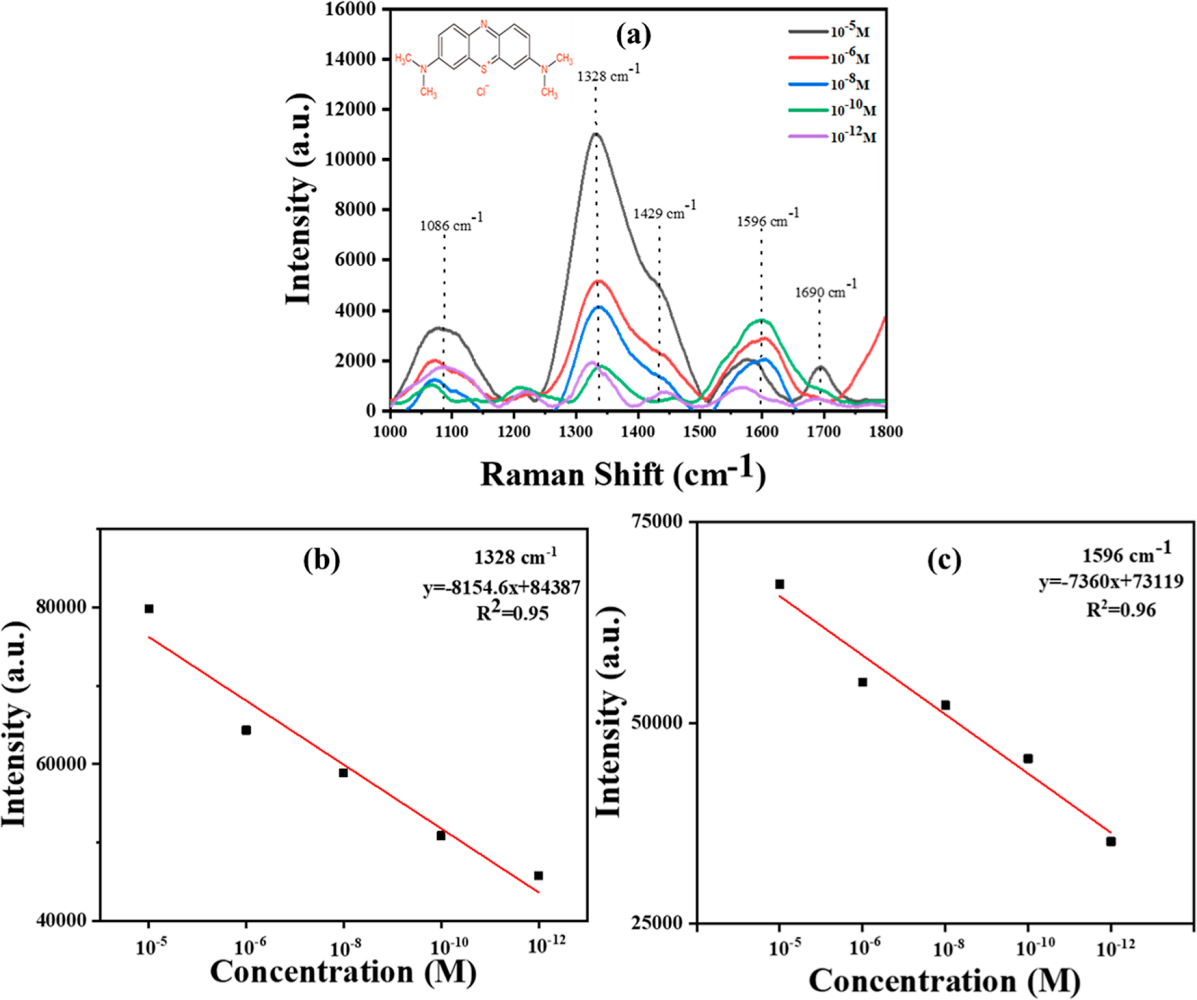
(a) SERS spectra at various concentrations of methylene blue adsorbed on the 15 AZ sample. (b,c) Linear fit of the peak at 1328 and 1596 cm^–1^ versus methylene blue concentrations for the 15 AZ sample.

Further, to check the sensitivity, the ZnO/Ag nanohybrid (15 AZ) SERS substrate was taken and tested at different methylene blue concentrations of 1 × 10^–5^,1 × 10^–6^, 1 × 10^–8^, 1 × 10^–10^, and 1 × 10^–12^ M. As shown in Figure 4a, as the concentration of methylene blue decreased from 1 × 10^–5^ to 1 × 10^–12^ M, the intensity of the spectra steadily decreased. Highly distinct and complete Raman peaks were observed up to a 10^–12^ M methylene blue concentration, as reported in the literature (Table S2). It was seen that with the decrease in MB concentration, there was a slight change in the peak position, and the intensity of some peaks increased or decreased, which can be explained on the basis of the molecular orientation of the dye molecules.^58,59^ Similar results were reported by Sarycheva et al.^60^ during SERS measurements of methylene blue by using two-dimensional titanium carbide MXene as the SERS substrate with a detection limit of 10^–7^ M. The detection limit of the present work of the 15 AZ sample for methylene blue molecules is at a very low concentration of 1 × 10^–12^ M. The intensity of the peak at 1328 cm^–1^ remained significant and distinguishable at low methylene blue concentrations. The graph between the intensity of the peaks versus the concentration of methylene blue for 15 AZ substrates at 1328 and 1596 cm^–1^ is illustrated in Figure 4b,c. The polarization of the excitation laser at the rough ZnO/Ag nanohybrid surface raises the detection limit of the molecular probes. The orientation of the incident light’s electric field with respect to the sample surface or molecular vibrations is referred to as the excitation laser’s polarization. When the electromagnetic field oscillates mostly in one direction, it is said to be polarized by the excitation laser. The intensity of Raman scattering can be strongly influenced by the orientation of this polarization. This outcome is consistent with some previous reports,^36,61,62^ as mentioned in Table 1.

**Table 1:** Detection Limit of the Ag/ZnO-Based SERS Substrate

SERS substrate	analyte	detection limit (M)	enhancement factor	refs
Ag/ZnO microspheres	methylene blue	10^–9^		(63)
ZnO nanoplates/Ag nanoparticles	methylene blue	10^–9^	6.2 × 10^6^	(36)
Ag/ZnO nanoscale villi	crystal violet	10^–13^	2.6 × 10^11^	(16)
Au@ZnO nanorods	methylene blue	10^–9^		(64)
coral-like nano Ag/ZnO	4-amino thiophenol	10^–10^		(65)
ZnO/Ag nanocomposites	rhodamine, crystal violet and methylene blue	10^–8^	5.04 × 10^7^	(66)
ZnO nanorods/Ag nanoflowers	crystal violet	10^–13^		(23)
Ag/ZnO nanorods	rhodamine 6G	10^–13^	2.7 × 10^8^	(24)
Ag/ZnO nanorods	rhodamine	10^–11^	9.2 × 10^8^	(25)
**ZnO/Agnanohybrid**	**methylene blue**	**10**^**–12**^	**3.7×10**^**7**^	**this work**

Another key parameter for SERS is the EF calculation. The peak at 1622.50 cm^–1^ for methylene blue is employed to calculate the EF of the 15 AZ sample using the following eq 1.

The enhancement factor was calculated according to the following equation^22,36^
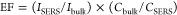
1

*C*_SERS_ and *C*_bulk_ are the detection limits of the methylene blue solution in the SERS-active 15 AZ and liquid pool, which are 10^–12^ and 2 × 10^–5^ M, respectively. *I*_SERS_ and *I*_Bulk_ are the measured SERS and normal Raman scattering intensities, respectively. We used the strongest signature stretching modes for *I*_SERS_ and *I*_Bulk_ at 1622.50 cm^–1^, which are 1672.32 and 901.20, respectively. The calculated EF value of the SERS-active 15 AZ is 3.7 × 10^7^, as per the literature calculations.^16,36,67^ These values are consistent with some previous reports, as mentioned in Table 1.

Figure S8 showed the long-term stability of a 15 AZ (ZnO/Ag) nanohybrid sample at room temperature over a period of 1 year. The structural, optical stability, and SERS were also studied by performing Raman, UV, XPS, and SERS investigations. Figure S8a shows the Raman spectra of the 15 AZ sample before and after 1 year. No change in Raman peak position or intensity was observed after 1 year. Similarly, no significant change in its absorption peak was observed after 1 year of storage (Figure S8b). Furthermore, Figure S8c shows the XPS spectrum of the 15 AZ sample, and no shifting was seen in the binding energy of Ag peaks after 1 year. It indicates that there was no surface oxidation of Ag NCs. To check the stability of the SERS activity of 15 AZ, the SERS experiment after 1 year was repeated under similar conditions (Figure S8d). It was observed that there was no significant change in enhancement of SERS peaks after 1 year, which corresponds to the excellent long-term stability of the 15 AZ sample.

### 2.3 Mechanism of High-Performance SERS Detection

The SERS mechanism of ZnO/Ag nanohybrid can be explained by two basic phenomena, such as EM enhancement due to LSPR of Ag NCs and charge transfer processes between ZnO/Ag nanohybrid and methylene blue molecules. The latter one is responsible for the SERS enhancement in the 0 AZ sample. The host ZnO NPs also show LSPR that is positioned in the near-infrared range, in contrast to noble metal NPs. The excitation wavelength of the laser employed was 514 nm (2.4 eV), which was far from the LSPR of ZnO. The laser is enough to excite the electrons from the VB of ZnO, transfer them to the LUMO of the methylene blue molecule, and polarize them. Therefore, the main contributor to SERS enhancement in the 0 AZ sample is the charge transfer mechanism.^10^

Furthermore, the SERS mechanism of the ZnO/Ag nanohybrid (except the 0 AZ sample) is mainly attributed to the strong LSPR of the Ag NCs and the strong chemical bonding between the Ag NCs and the host ZnO NPs with the analyte molecule. The LSPR of the Ag NCs was responsible for the enhancement of the Raman scattering of the host ZnO NPs due to the increased electric field between the Ag NCs, creating hotspots. Furthermore, it is now widely acknowledged that the spacing between metal nanoparticles, which is shown to result in SERS “hot spots” plays a significant role in SERS enhancement, when the average interparticle distance is smaller than 10 nm.^20,21^ As reported in HRTEM images in Figure S3a,b, the density of “hot spots” increases noticeably as the Ag NCs concentration increases. When switching from the no “hot spot” situation (0 AZ sample) to the “hot spot” condition (up to 15 AZ sample), the high density of hot spots appears to be the cause of the large SERS enhancement.^20^ But at a higher Ag concentration, i.e., a 20 AZ sample, the SERS activity decreases significantly. The reason behind this decrease is the quantum mechanical effects, which become more significant and cause the electromagnetic enhancement to decrease as the gap size is reduced to the subnanometer level.^68,69^

**Figure 5:**
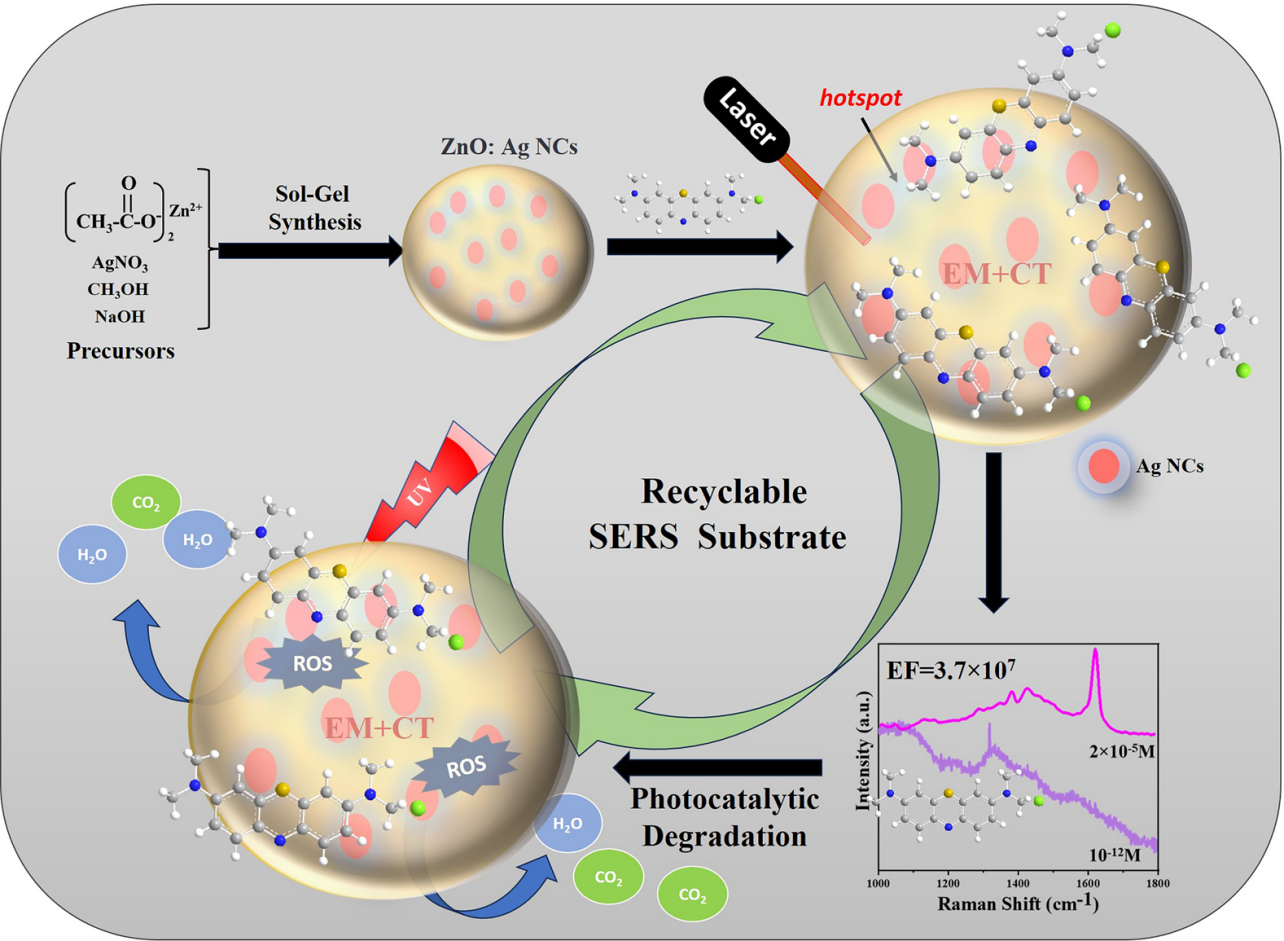
Schematic of SERS enhancement using the ZnO/Ag nanohybrid.

As indicated by Figure S2a,b and the prior analysis, the 0 AZ and 15 AZ samples show a porous nature. As a result, more analyte molecules will enter the nanopores and approach the Ag NCs. In order to increase the Raman signal, more molecules are excited within the range of the excitation light spot as compared to within the 0 AZ substrate. The strong local electromagnetic field on the Ag NCs surface or due to the hot spot causes the analyte molecules to produce a greater Raman scattering effect.^49−51^ Additionally, the strong chemical bonding between the Ag NCs and the host ZnO NPs facilitates charge transfer from the Ag NCs to the host ZnO NPs and thus increases the Raman scattering efficiency. The combination of these two effects leads to SERS enhancement of the host ZnO NPs embedded with Ag NCs. Also, the ZnO/Ag nanohybrid system shows recyclable SERS activity under the effect of UV light irradiation; this system serves as a recyclable SERS substrate due to the synergetic effect of host ZnO and Ag counterparts. In brief, Figure 5 schematically describes the synthesis of ZnO/Ag nanohybrid synthesis via the sol–gel method and various other phenomena, including SERS detection, sensitivity, and photodegradation-based self-cleaning ability, that result in the recyclable SERS substrate.

## 3 Conclusions

The present work demonstrates that highly stable, sensitive, and recyclable ZnO/Ag nanohybrids have great potential for long-term SERS applications. The nanohybrid structures are made of host ZnO NPs embedded with Ag NCs. The ZnO/Ag nanohybrid provided an effective platform for enhancing the SERS signal due to the formation of hotspots. At optimized conditions, these hotspots in the ZnO/Ag nanohybrid allow the detection of target molecules at very low concentrations (10^–12^ M). The SERS enhancement factor was found to be around 3.7 × 10^7^. The ZnO/Ag nanohybrid shows reproducibility with a good RSD value of 8.59%. Furthermore, their recyclability makes them extremely cost-effective and suitable for real-time monitoring; the ZnO/Ag nanohybrid lost only 10.19% SERS activity after 4 cycles of reuse. For long-term stability (1 year), these nanohybrids show no significant change in their characteristics, suggesting their high long-term stability. Therefore, these ZnO/Ag nanohybrid are promising candidates for the development of highly sensitive SERS-based sensors for environmental monitoring and can be applied in the cross-disciplinary fields of biodetection and disease diagnosis.

## 4 Experimental Details

### 4.1 Materials Required

Zinc acetate dihydrate [(CH_3_COO)_2_Zn·2H_2_O, 98.5%, SRL], methanol (99.8%, SRL), silver nitrate (AgNO_3_, ≥99.0%, Sigma-Aldrich), sodium hydroxide (NaOH, 98%, SRL), methylene blue (Alfa Aesar C_16_H_18_CIN_3_S), and DI water (HPLC grade, Merck) were used.

### 4.2 Synthesis of Pure and Ag-Embedded ZnO

**Figure 6:**
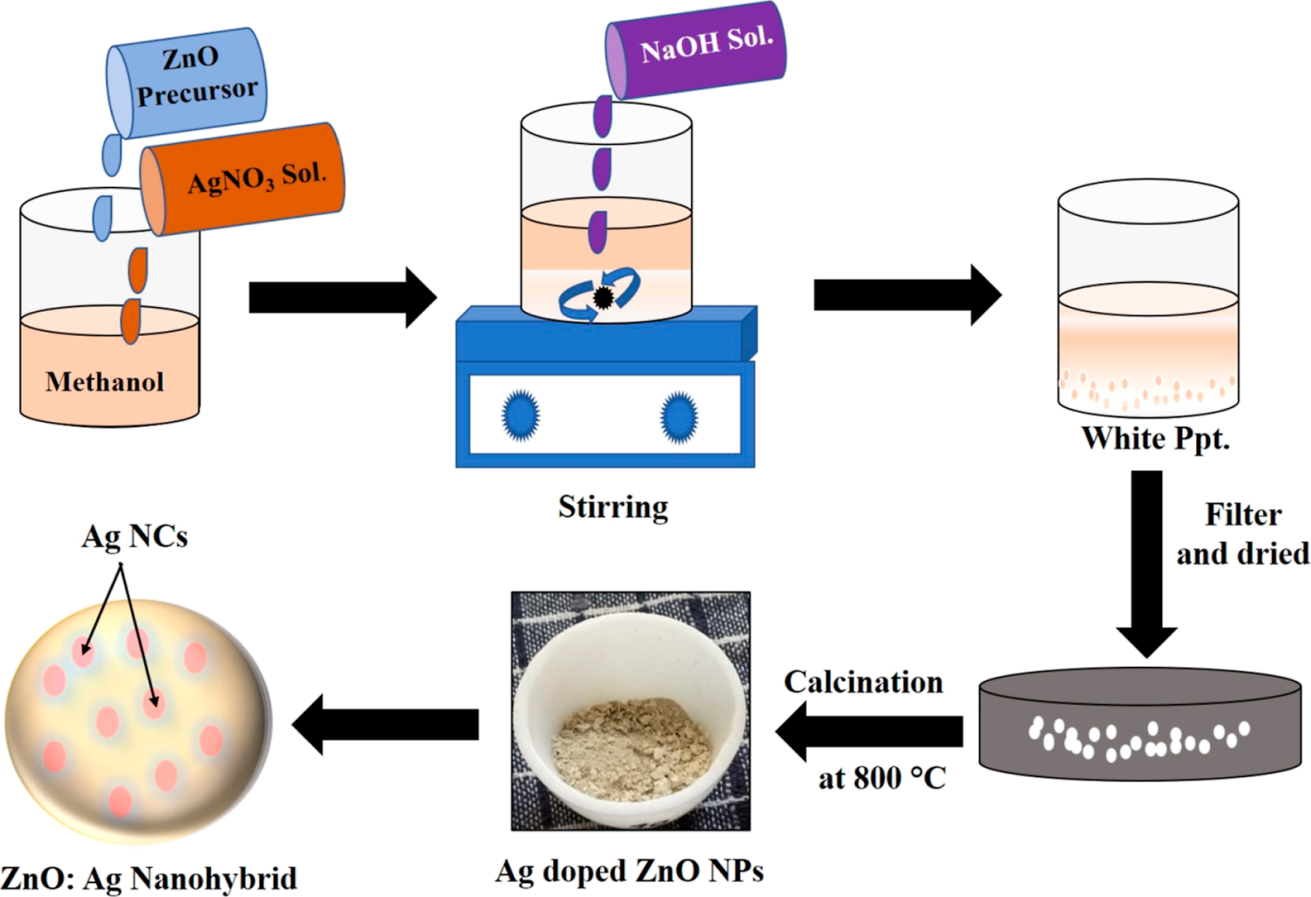
Schematic showing the sol–gel synthesis of the ZnO/Ag nanohybrid.

The Sol–gel method was used to synthesize ZnO NPs with embedded Ag NCs, i.e., ZnO/Ag nanohybrids, as shown in Figure 6. Methanol (30 mL of methanol) and zinc acetate dihydrate (4 g) were combined, and the mixture was stirred for 5 min at room temperature using a magnetic stirrer at 450 rpm. Six different amounts of AgNO_3_ were added according to the calculations (0, 1, 5, 10, 15, and 20 mol %), and a homogeneous mixture was prepared. The second solution was prepared by combining 16 g of NaOH with 20 mL of methanol. Both solutions were mixed under constant stirring for up to 10 min until white precipitates were formed. The precipitate was filtered, washed several times with methanol, and dried overnight at room temperature. For complete drying, the samples were kept in an oven at 100 °C for 10–11 h and then calcined in a muffle furnace for 3 h at 800 °C. The resulting ZnO/Ag nanohybrid was then gently crushed in a mortar and pestle after cooling to room temperature. The chemical reaction that occurred during synthesis is given in eqs 2–4
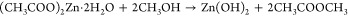
2
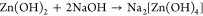
3
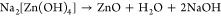
4

### 4.3 Characterizations

The crystalline structure was analyzed with an X-ray diffractometer (XRD) (PAN analytical X’pert PRO) with Cu Kα. The X-ray photoelectron spectroscopy (XPS) analysis was carried out with a PHI 5000 Versaprobe-Scanning XPS Microprobe. The transmission electron microscopy (TEM) and high-resolution transmission electron microscopy (HRTEM) images were captured using FEI Tecnai TF20. The ultraviolet–visible (UV–vis) spectra were recorded in a spectrophotometer (double beam LI-2800). Thermogravimetric analysis (TGA) was done by an EXSTAR Model-SII 6300. Fourier transform infrared (FTIR) data were recorded in an IRAffinity-1SWL spectrophotometer. Brunauer–Emmett–Teller (BET) analysis was performed on quantachrome instruments (Autosorb iQ Station 1), and the specific surface areas were determined by the BET method. Raman spectra were studied using a 514 nm laser beam with a WiTec Raman spectrometer in the 50–1800 cm^–1^ range. 0.5 mW of Raman laser energy was directed toward the sample surface. The SERS spectrum integration time is 10 s. The laser beam spot diameter in the samples was around 25 μm.

### 4.4 SERS Measurement and Recyclability Tests

The SERS experiment was performed on a ZnO/Ag nanohybrid (0–20 AZ samples) with methylene blue dye molecules, serving as the model analyte molecule. First, 5 mg of each ZnO/Ag nanohybrid was dispersed in 5 mL of a 20 μM aqueous methylene blue solution. The mixture was then stirred continuously for 2 h. A drop of this solution was placed on a glass slide and dried at room temperature. The Raman spectra were recorded under ambient conditions at several spots, and the final data were obtained by taking an average of those obtained in all cases. Furthermore, SERS measurements with lower concentrations of methylene blue solutions, such as 1 × 10^–5^, 1 × 10^–6^, 1 × 10^–8^, 1 × 10^–10^, and 1 × 10^–12^ M, were repeated. All spectra were obtained by averaging the spectra of five different areas of the substrates.

The following procedures were used to evaluate the recyclability: following the detection of SERS, the ZnO/Ag nanohybrid substrate was directly exposed to UV light (385 nm) for 120 min. The degradation level of methylene blue was determined by using the substrates. Finally, the substrates were again employed and submerged in an aqueous solution of methylene blue for SERS detection. This process was repeated for up to four cycles.

## Supporting Information Available

XRD spectra of different concentrations of Ag/ZnO; TEM and HRTEM images of Ag/ZnO; EDX mapping of 0 and 15 AZ; FTIR, TGA, and Raman spectra of Ag/ZnO; normal Raman spectra of MB powder and aqueous; RAMAN and UV spectra showing long-term stability of Ag/ZnO; and average Ag NCs size estimated by using Mie theory calculations with increasing Ag concentration (PDF)
