# Supplementary material for: Sensitive, Stable, and Recyclable ZnO/Ag Nanohybrid Substrates for Surface-Enhanced Raman Scattering Metrology
Source: ACS Mater Au. 2024 Apr 10;4(4):413–23. doi: 10.1021/acsmaterialsau.4c00002 (PMC11240408; doi:10.1021/acsmaterialsau.4c00002)
Supplement: Supplementary file 1 — mg4c00002_si_001.pdf [file mg4c00002_si_001.pdf]

Supplementary Information

Sensitive, Stable, and Recyclable ZnO:Ag Nanohybrid Substrates for Surface-Enhanced Raman Scattering Metrology

Samriti Sharma[a], Promod Kumar[b], A.Yu. Kuznetsov *[c], H. C. Swart[b], Jai Prakash*[a]

[a]Department of Chemistry, National Institute of Technology Hamirpur, Hamirpur (H.P.)-177005, India.

[b]Department of Physics, University of the Free State, Bloemfontein 9301, Republic of South Africa.

[c]Department of Physics, Centre for Materials Science and Nanotechnology, University of Oslo, N-0316, Oslo, Norway.

***Corresponding author**: jaip@nith.ac.in, andrej.kuznetsov@fys.uio.no

Figure S1 XRD spectra of different concentrations of Ag:ZnO.

Figure S2 TEM images of Ag:ZnO.

Figure S3 HRTEM images of Ag:ZnO.

Figure S4 The Particle size distribution curve formed from HRTEM images of 10AZ, 15AZ, and 20AZ samples of ZnO:Ag nanohybrid.

Figure S5 Shows the FESEM and EDX mapping images of 0AZ and 15AZ.

Figure S6 FTIR, TGA and Raman spectra of Ag:ZnO.

Figure S7 Normal Raman spectra of MB in powder form and MB in aqueous solution

Figure S8 RAMAN, UV spectra showing long term stability of Ag:ZnO.

Table S1: Average Ag NCs size estimated by using Mie theory calculations with increasing Ag concentration.

Table S2. Normal and SERS Raman shifts and their assignments for MB molecules.

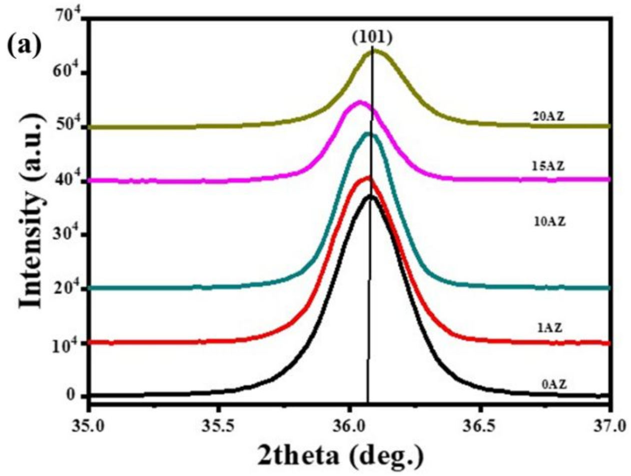

Figure S1 (a) XRD spectra of ZnO:Ag nanohybrid showing a shift in (101) peak with Ag doping.

Table S1: Average Ag NCs size estimated by using Mie theory calculations with increasing Ag content:

Sample details	Temperature (°C)	SPR wavelength (nm)	FWHM (eV)	Cluster size (nm)
1AZ	800	471	0.79	2.3
5AZ	800	478	0.61	3
10AZ	800	482	0.52	3.5
15AZ	800	485	1.29	1.4
20AZ	800	492	1.19	1.5

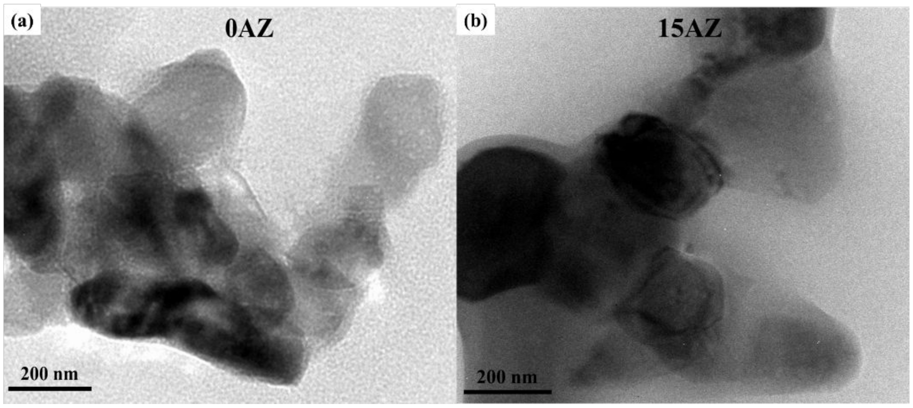

Figure S2(a-b) TEM images of 0AZ and 15AZ samples of ZnO:Ag nanohybrids.

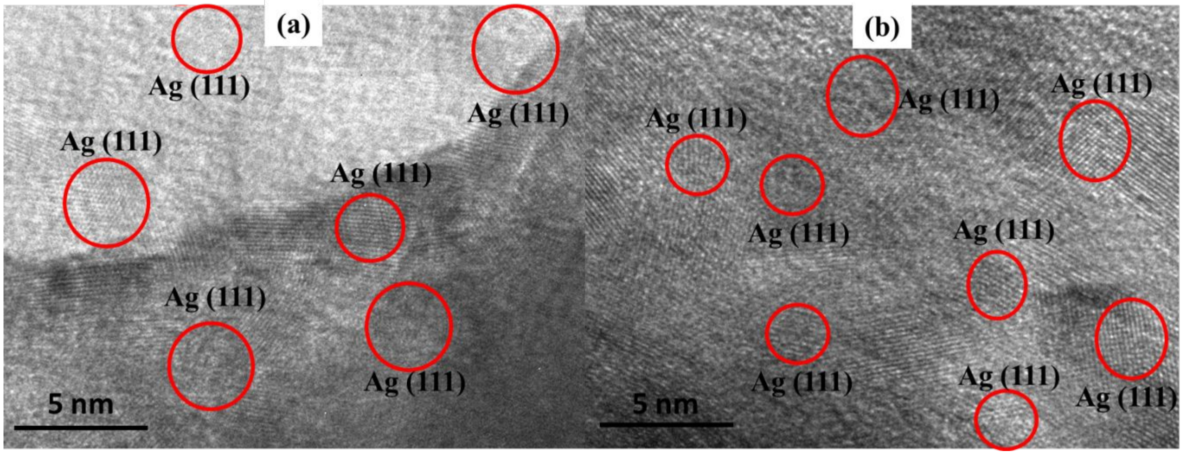

Figure S3 (a-b) HRTEM images of 10AZ and 20AZ samples of ZnO:Ag nanohybrid.

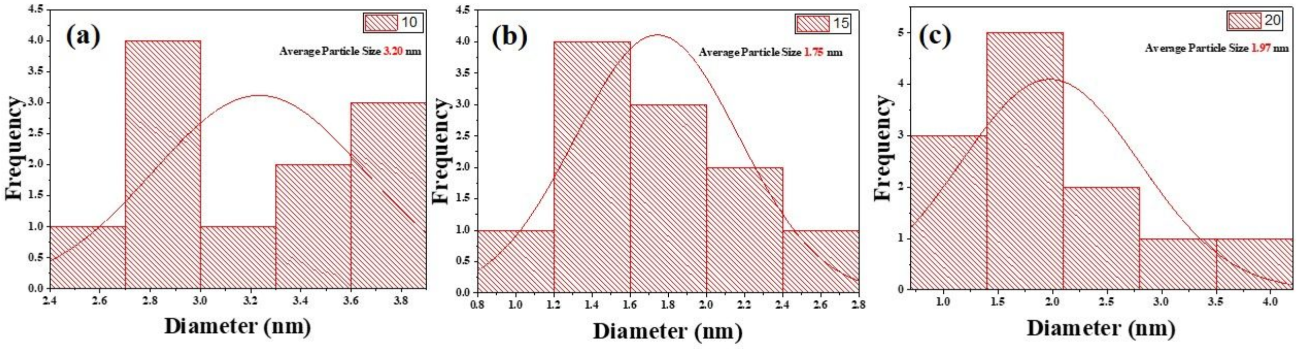

Figure S4 (a-c) The Particle size distribution curve formed from HRTEM images of 10AZ, 15AZ, and 20AZ samples of ZnO:Ag nanohybrid.

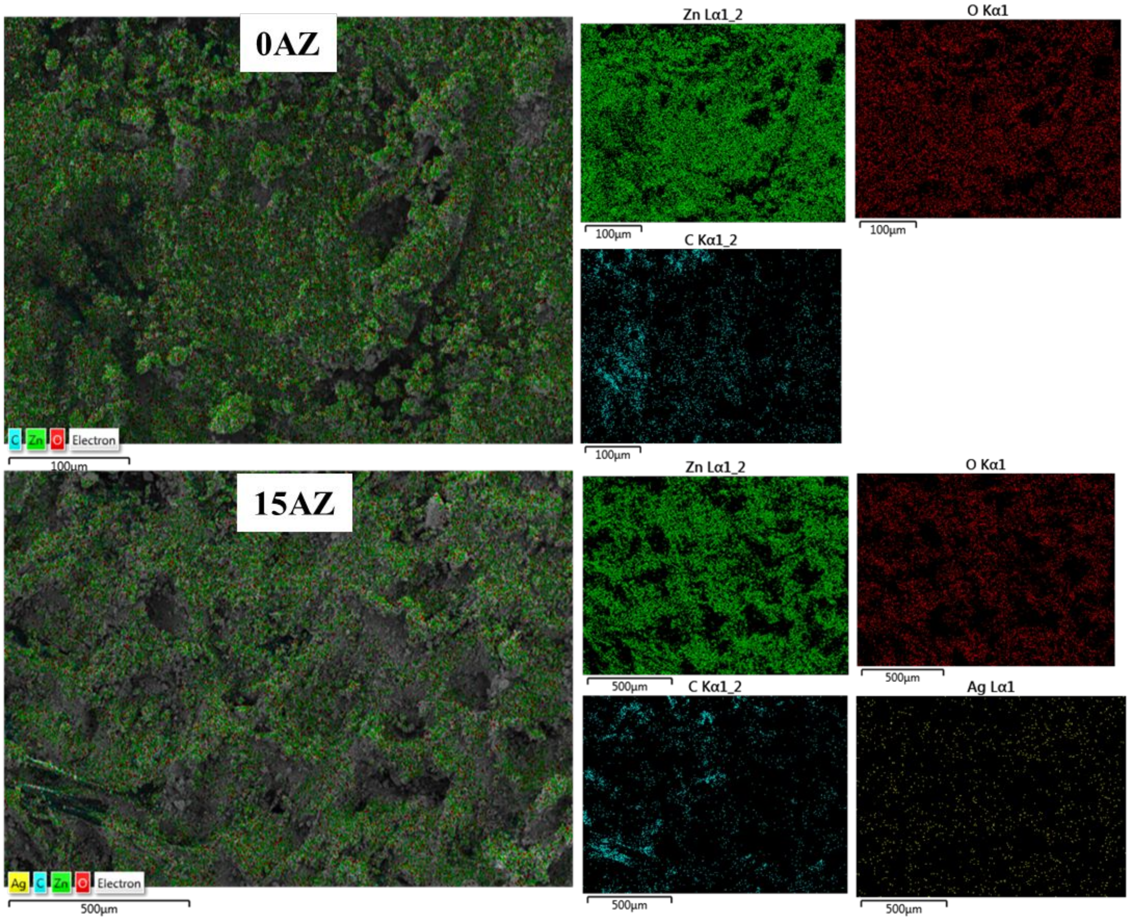

Figure S5 -FESEM the EDX mapping images of 0AZ and 15AZ.

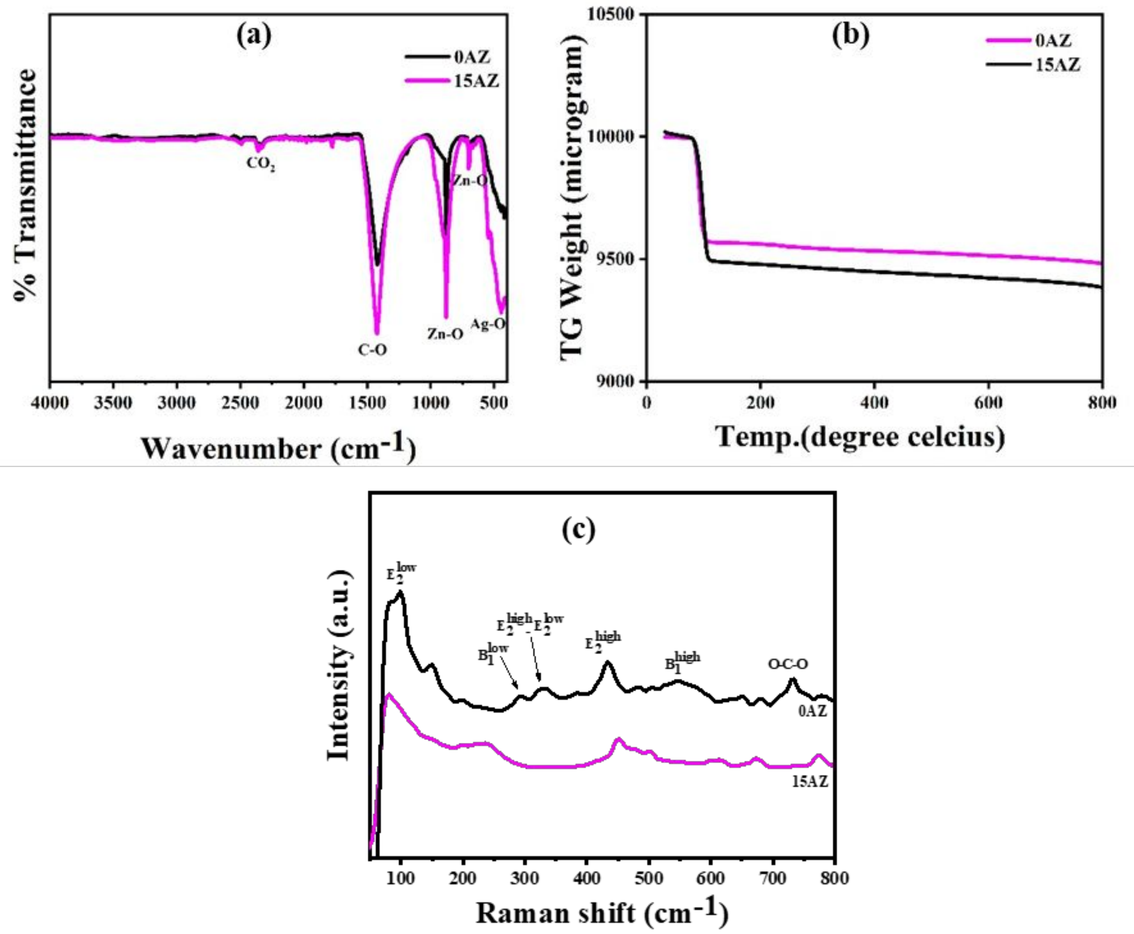

Figure S6 (a) FTIR Spectra of 0AZ and 15AZ samples**. (b)** TGA of 0AZ and 15AZ samples. **(c)** Raman spectra of 0AZ and 15AZ samples.

FT-IR spectra of ZnO: Ag nanohybrid (0AZ and 15AZ) are shown in **Figure S6(a)**. All samples exhibit an absorption band at 2360 cm^{-1} as a result of the ambient CO_2 present at the time of observation [1, 2]. The Peak at 1420 cm^{-1} is corresponds to the C-H stretching vibration [3]. Peaks at 834 cm^{-1} and 702 cm^{-1} are attributed to Zn-O modes and the peak at 459 cm^{-1} attributed to Ag-O mode. These data confirms, that 15AZ NPs exhibit higher doping properties in the Zn-O vibrational mode.[3] The TGA graph of ZnO: Ag NCs are shown in **Figure S6(b).** It has been discovered that weight loss occurred with the temperature increase from room temperature to 109 °C as a result of water desorption and solvent elimination. Due to the inclusion of Ag ions in

the ZnO crystal lattice, the weight loss is less for 15 AZ than for 0AZ. The TGA study confirms the XRD findings that the calcined ZnO: Ag NCs were carbon-free.

Figure S6(c) shows the Raman spectra of the Ag:ZnO nanohybrid within the spectral range between 100 -800 cm^{-1}. Peaks around 97 and 433 cm^{-1} in both samples corresponds to E_2(low) and E_2(high) fundamental phonon mode of ZnO. These are the characteristic modes of ZnO on Ag doping they become broader[4]. In addition, peaks at 289 and 547 cm^{-1} are the salient modes referred as B_1^{low} and B_1^{high} respectively. Furthermore, it is believed that the O-C-O symmetric bends in the acetate groups utilised as reactants throughout the synthesis process are the source of the Raman mode detected at 731 cm^{-1}. Similar, results were reported in literature[4, 5].

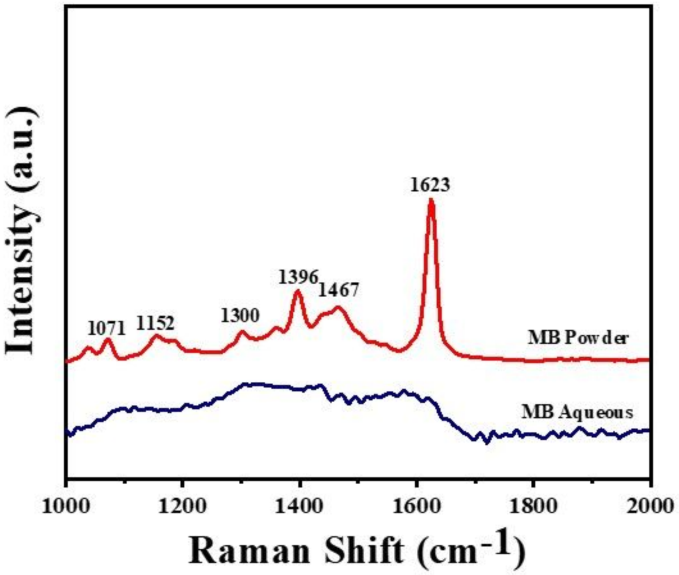

Figure S7: Normal Raman spectra of MB in powder form and MB in aqueous solution**.**

Table S2. Normal and SERS Raman shifts and their assignments for MB molecules [2, 6, 7].

Normal MB	Measured SERS MB	Raman peak assignments
1070 cm^{-1}	1086 cm^{-1}	Symmetric C-N stretching
1301 cm^{-1}	1328 cm^{-1}	C-C ring stretching
1469 cm^{-1}	1429 cm^{-1}	C-C ring stretching
1622 cm^{-1}	1596 cm^{-1}	C-C (and C-N-C) ring stretching

Enhancement Factor Calculation :

The SERS enhancement factor (EF) is given by:

$EF= N_{Normal}\ I_{SERS}/N_{SERS}\ I_{Bulk}$

where N_{Normal} and N_{SERS} are the number of molecules probed in the aqueous sample and on the SERS substrates, respectively. I_{SERS} and I_{Bulk} are the corresponding normal Raman and SERS intensities.

$$N_{Normal} = \Pi r^2 h \times \rho \times N_A/M$$

where πr^2 h is the optical excitation volume; ρ is the density of MB dye (1.23 g/cm^3) in the bulk; N_A is the Avogadro number, and M is the molecular weight of MB molecule (319.85 g/mol).

The laser spot volume was determined by multiplying its (πr^2, $r=0.61\times\lambda/N.A.$) and depth of focus ($h=2\lambda/(N.A.)^2$), where $\lambda=514$ nm is the excitation wavelength and N.A.=0.5 is the numerical

aperture[8, 9]. The number of molecules being probed (N_{Normal}) was calculated to be 0.028 x 10^{13}. The value of N_{SERS} was obtained by dividing the laser surface area (6.28 μm^2) by the effective cross-sectional area per molecule (6×10^{-7}). The calculated value of N_{SERS} to obtain the number of molecules on Ag:ZnO substrate are 1.04×10^7 [10, 11]. We used the strongest signature stretching modes for I_{SERS} and I_{Bulk} at 1622.50 cm^{-1}, which are 1672.32 and 901.20 respectively. The calculated EF value of the SERS-active 15AZ is 3.3×10^6.

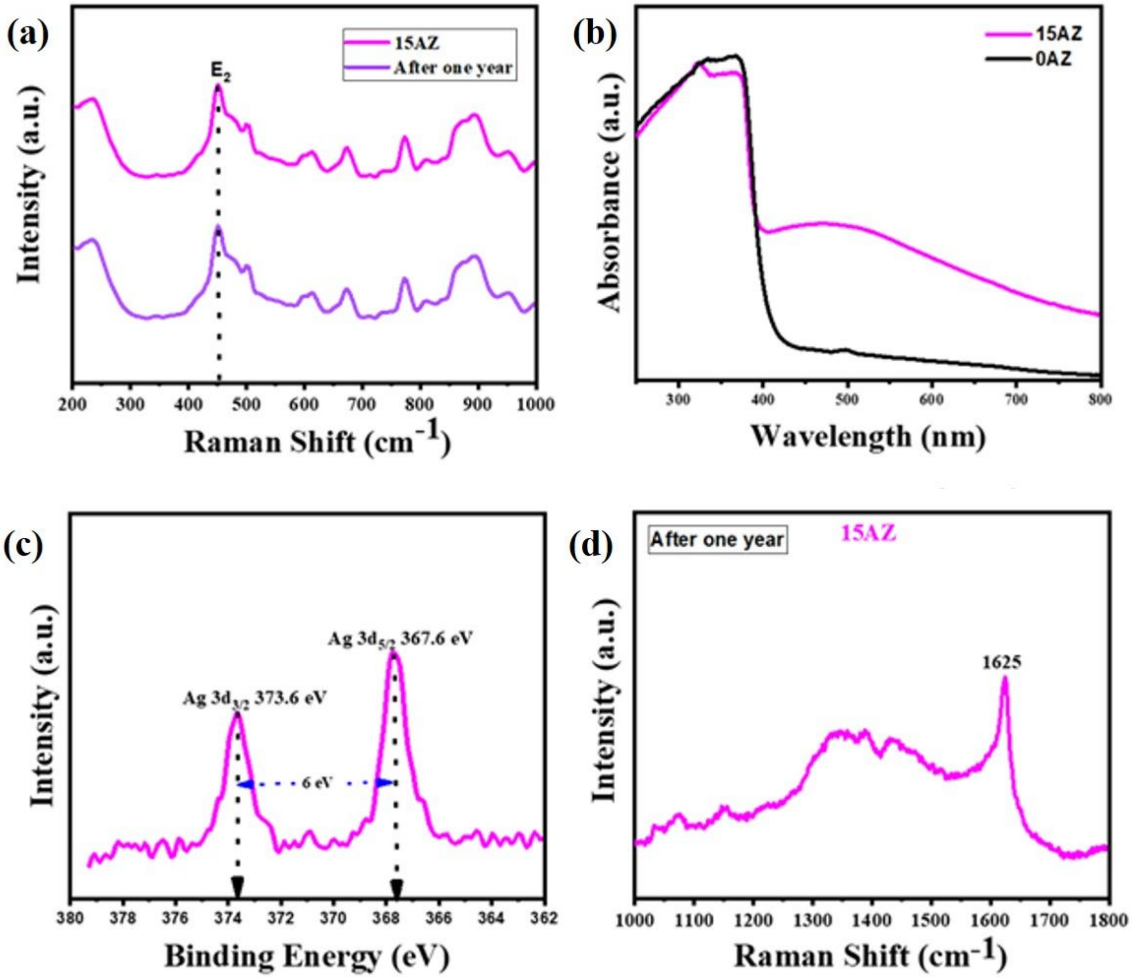

Figure S8. Measurements showing the long term stability of ZnO:Ag nanohybrid after 1 year of storage at room temperature: **(a)** Raman **(b)** Optical absorption spectra **(c)** XPS and **(d)** SERS spectra.

References:

1. Jarvin, M.; Kumar, S. A.; Rosaline, D. R.; Foletto, E. L.; Dotto, G. L.; Inbanathan, S. S. R., Remarkable sunlight-driven photocatalytic performance of Ag-doped ZnO nanoparticles prepared by green synthesis for degradation of emerging pollutants in water. *Environmental Science and Pollution Research* **2022,** *29* (38), 57330-57344.
2. Singh, N.; Prakash, J.; Misra, M.; Sharma, A.; Gupta, R. K., Dual functional Ta-doped electrospun TiO2 nanofibers with enhanced photocatalysis and SERS detection for organic compounds. *ACS applied materials interfaces*

2017, *9* (34), 28495-28507.
3. Vivek, C.; Balraj, B.; Thangavel, S., Structural, optical and electrical behavior of ZnO@Ag core–shell nanocomposite synthesized via novel plasmon-green mediated approach. *Journal of Materials Science: Materials in Electronics* **2019,** *30* (12), 11220-11230.
4. Postica, V.; Vahl, A.; Santos-Carballal, D.; Dankwort, T.; Kienle, L.; Hoppe, M.; Cadi-Essadek, A.; de Leeuw, N. H.; Terasa, M.-I.; Adelung, R.; Faupel, F.; Lupan, O., Tuning ZnO Sensors Reactivity toward Volatile Organic Compounds via Ag Doping and Nanoparticle Functionalization. *ACS Applied Materials & Interfaces* **2019,** *11* (34), 31452-31466.
5. Musa, I.; Qamhieh, N.; Mahmoud, S. T., Synthesis and length dependent photoluminescence property of zinc oxide nanorods. *Results in Physics* **2017,** *7*, 3552-3556.
6. Vu, X. H.; Dien, N. D.; Ha Pham, T. T.; Trang, T. T.; Ca, N. X.; Tho, P. T.; Vinh, N. D.; Van Do, P., The sensitive detection of methylene blue using silver nanodecahedra prepared through a photochemical route. *RSC Advances* **2020,** *10* (64), 38974-38988.
7. Ha Pham, T. T.; Vu, X. H.; Dien, N. D.; Trang, T. T.; Kim Chi, T. T.; Phuong, P. H.; Nghia, N. T., Ag nanoparticles on ZnO nanoplates as a hybrid SERS-active substrate for trace detection of methylene blue. *RSC Advances* **2022,** *12* (13), 7850-7863.
8. Tao, C.-a.; An, Q.; Zhu, W.; Yang, H.; Li, W.; Lin, C.; Xu, D.; Li, G., Cucurbit[n]urils as a SERS hot-spot nanocontainer through bridging gold nanoparticles. *Chemical Communications* **2011,** *47* (35), 9867-9869.
9. Gopinath, A.; Boriskina, S. V.; Reinhard, B. M.; Dal Negro, L., Deterministic aperiodic arrays of metal nanoparticles for surface-enhanced Raman scattering (SERS). *Optics Express*

2009, *17* (5), 3741-3753.
10. Ganesh, S.; Venkatakrishnan, K.; Tan, B., Quantum scale organic semiconductors for SERS detection of DNA methylation and gene expression. *Nature Communications* **2020,** *11* (1), 1135.
11. Villaeys, A.; Zouari, M., Role of the substrate field inhomogeneities in coherent resonant raman scattering. *The Journal of Physical Chemistry A*

2007, *111* (38), 9522-9531.
